# Sample size implications of mortality definitions in sepsis: a retrospective cohort study

**DOI:** 10.1186/s13063-018-2570-2

**Published:** 2018-03-27

**Authors:** Sushant Govindan, Hallie C. Prescott, Vineet Chopra, Theodore J. Iwashyna

**Affiliations:** 1 Department of Medicine, University of Michigan Health System, Ann Arbor, MI USA; 2 Center for Clinical Management Research, Ann Arbor VA Healthcare System, Ann Arbor, MI USA; 3 Patient Safety Enhancement Program, Ann Arbor VA Healthcare System, Ann Arbor, MI USA; 4 Taubman Center, FL 3 Rm 3920, 1500 E Medical Center Dr SPC 5360, Ann Arbor, MI 48109 USA

**Keywords:** Sepsis, Randomized clinical trials, Sample size, Methods, Outcomes

## Abstract

**Background:**

Many randomized controlled trials (RCTs) employ mortality at a given time as a primary outcome. There are at least three common ways to measure 90-day mortality: first, all-location mortality, that is, all-cause mortality within 90 days of randomization at any location. Second, ARDSnet mortality is death in a healthcare facility of greater intensity than the patient was in prior to the hospitalization during which they were randomized. Finally, in-hospital mortality is death prior to discharge from the primary hospitalization of randomization. Data comparing the impact of these different measurements on sample size are lacking. We evaluated the extent to which event rates vary by mortality definition.

**Methods:**

This was a retrospective cohort study of 30,691 patients hospitalized at Veterans Affairs (VA) hospitals for sepsis during 2009. 12,727 (41.5%) received care in an ICU setting. For each patient, we measured event rates for three different 90-day mortality outcomes: all-location mortality, ARDSnet mortality, and in-hospital mortality. We also calculated sample sizes necessary to power an example RCT given those event rates.

**Results:**

At 90 days, all-location mortality was 26.4% (95% CI 25.9–26.9%), ARDSnet mortality was 19.2% (95% CI 18.8–19.7%), and in-hospital mortality was 13.4% (95% CI 13.0–13.8%) (*p* < 0.01 all comparisons). These respective event rates result in different required sample sizes to achieve a 20% relative reduction in mortality with 80% power and a 5% false positive rate. Such a trial of VA sepsis patients would require 2080 patients for all-location mortality, 3080 for ARDSnet mortality, and 4796 for in-hospital mortality. Among sepsis patients mechanically ventilated in an ICU, 2438 experienced all-location mortality (46.2% [95% CI 44.8–47.5%]), 2181 experienced ARDSnet mortality (41.3% [95% CI 40.0–42.6%]), and 1894 experienced in-hospital mortality (36.0% [95% CI 34.7–37.3%]).

**Conclusions:**

Event rates vary substantially in sepsis patients based on the chosen 90-day mortality definition. This could have important implications for RCT design trade-offs.

**Electronic supplementary material:**

The online version of this article (10.1186/s13063-018-2570-2) contains supplementary material, which is available to authorized users.

## Background

Patient recruitment and outcome ascertainment are key components of randomized controlled trials (RCTs) [1]. In the context of critically ill patients with sepsis, mortality is a common outcome chosen given the high event rates in this patient population [2]. However, there are multiple ways to define mortality as an outcome. With limited budgets, trialists make critical trade-offs in allocating resources to patient recruitment to achieve adequate power and meet follow-up requirements for any particular chosen outcome [1, 3].

One strategy to improve efficiency is choosing a trial outcome that optimally balances these issues. For example, consider 90-day mortality in critical care trials [4]. The outcome could be measured as 90-day all-location mortality (e.g., 6S trial) [5], which is all-cause mortality within 90 days of randomization at any location. Alternatively, it could be measured as ARDSnet mortality, or 90-day mortality in a healthcare facility of greater intensity than the patient was in prior to the hospitalization during which they were randomized (e.g., ARDSnet trials) [6]. ARDSnet mortality is a relevant outcome for sepsis patients given that over 60% suffer from ARDS, with resultant higher mortality compared to non-ARDS sepsis patients [7]. Finally, mortality could be measured as 90-day in-hospital mortality [4].

Given the largely negative RCTs in critical care concerning patients with sepsis, there have been calls to reassess the trial design process. One particular area of emphasis is how to define the chosen outcome [2, 8]. There are important implications for study design when deciding among mortality measures. Since 90-day all-location mortality captures deaths in all locations, it has the highest event rate. However, supplemental resources may be necessary to prevent loss to follow-up, including the hiring of additional personnel [3]. Alternatively, 90-day in-hospital mortality offers a less complicated approach because patient follow-up ceases at hospital discharge. However, the lower event rates demand larger sample sizes to achieve the same statistical power. ARDSnet mortality offers an event rate between that of the previous two outcomes. Methodological factors regarding outcome measurement also exist. These include the presence or absence of national health systems, as well as the availability of automated or electronic patient data for follow-up. Unfortunately, there is little published data to quantitatively inform the selection of mortality endpoints.

In an effort to better inform RCT design, quantifying how varying mortality measures affect study power is necessary. Therefore, we compared event rates and sample sizes between 90-day all-location, ARDSnet, and in-hospital mortality. We studied a cohort of patients hospitalized for sepsis—as sepsis effects mortality for at least 2 years [9]—and we performed stratified analyses to understand how severity of acute illness altered relative event rates.

## Methods

### Study population

This was a multi-center, retrospective cohort study of 32,680 patients hospitalized for sepsis in the US nationwide Veterans Affairs (VA) healthcare system (including more than 100 hospitals) during 2009. Sepsis hospitalizations were identified using the method of Angus et al. [10]. We excluded transfer-in patients from the analysis because of an unclear start time for their hospitalization. For patients who had multiple hospitalizations for sepsis in 2009, only the first hospitalization was included.

### Data sources and mortality endpoints

For each patient, we calculated three different mortality outcomes: 90-day all-location mortality, 90-day ARDSnet mortality, and 90-day in-hospital mortality. In order to calculate ARDSnet mortality, we ascertained each patient’s location prior to hospitalization and after discharge. We measured patient location using four data sources: (1) VA Inpatient Evaluation Center (IPEC) files on inpatient VA hospitalizations, (2) VA IPEC files on inpatient VA nursing homes, (3) MedPAR files, and (4) “fee-based” care files. The only patient care not in this database would be that which was paid for out of pocket or by Medicaid or private insurance, both of which are uncommon for patients enrolled in the VA system. There was no loss to follow-up regarding mortality given that the VA tracks patient mortality in both the inpatient and outpatient settings.

Collectively, these files capture all inpatient healthcare paid for or provided by the VA, as well as all non-VA care paid for by Medicare. We assumed that a patient’s location was at home for any day that he or she was known to be alive and not admitted to an inpatient healthcare facility based on the above files [11]. These files are also linked to national death records to ensure accurate mortality estimates. By ascertaining each patient’s daily location for the one year before and after sepsis hospitalization, we ensured that no healthcare use was “double-counted” [12]. The only patient care not in this database would be that which was paid for out of pocket or by private insurance not captured by Medicare. This database provides an ability to measure location of death and resource utilization [13], something that is extremely difficult to measure within private or Medicare claims [14].

### Outcomes

The primary outcome was the calculated sample size for each of the three mortality measures. All calculations were made to detect a 20% relative reduction in mortality, with 80% power and a 5% false positive rate. We used relative reduction because this measure of treatment efficacy is the least impacted by variation in event rates [15, 16]. We also assumed a non-time varying reduction in mortality because this is the standard practice for power calculations in trials employing 90-day mortality as an endpoint. Calculations were done for the entire sample and in the subgroup that used both the intensive care unit (ICU) and mechanical ventilation (MV). A secondary analysis was done with calculations to detect a 5% absolute reduction in mortality (Additional file 1).

### Statistical analysis

Cohort characteristics were analyzed as numbers (percentages), means (standard deviations [SD]), or medians (interquartile ranges [IQR]). The two-sample test of proportions assessed for differences between mortality event rates. Power calculations were done for each mortality outcome. We employed two-sided significance testing with a *p* value of less than 0.05 as significant, and we defined 95% confidence intervals (CI). We also compared the different mortality outcomes via survival analysis and Kaplan-Meier curves. The research was approved by the Ann Arbor VA Institutional Review Board. We used Stata MP version 14 for all analyses (StataCorp 2016, College Station, TX).

## Results

We identified 32,680 Veterans hospitalized for sepsis in 2009. After excluding 1989 inter-hospital transfers, the final sample contained 30,691 patients (Table 1). The mean age was 69.9 years (SD 12.0); 12,727 (41.5%) received care in an ICU setting, and 5280 (17.2%) received mechanical ventilation.

**Table 1:** Patient demographics (*N* = 30,691)

Age, years (SD)	69.9 (12.0)
Male, *N* (%)	29,753 (96.9)
Comorbidities, *N* (%)	
CHF	5960 (19.4)
PVD	1909 (6.2)
HTN	14,569 (47.8)
Chronic liver disease	2116 (6.9)
Malignancy	5948 (19.4)
Obesity	1293 (4.2)
Race, *N* (%)	
White/Caucasian	22,134 (72.1)
Black/African American	5650 (18.4)
Unknown	2483 (8.1)
Other	424 (1.4)
Hospital length of stay, days (SD)	11.5 (13.1)
ICU, *N* (%)	12,727 (41.5)
Mechanically ventilated, *N* (%)	5280 (17.2)

**Fig. 1:**
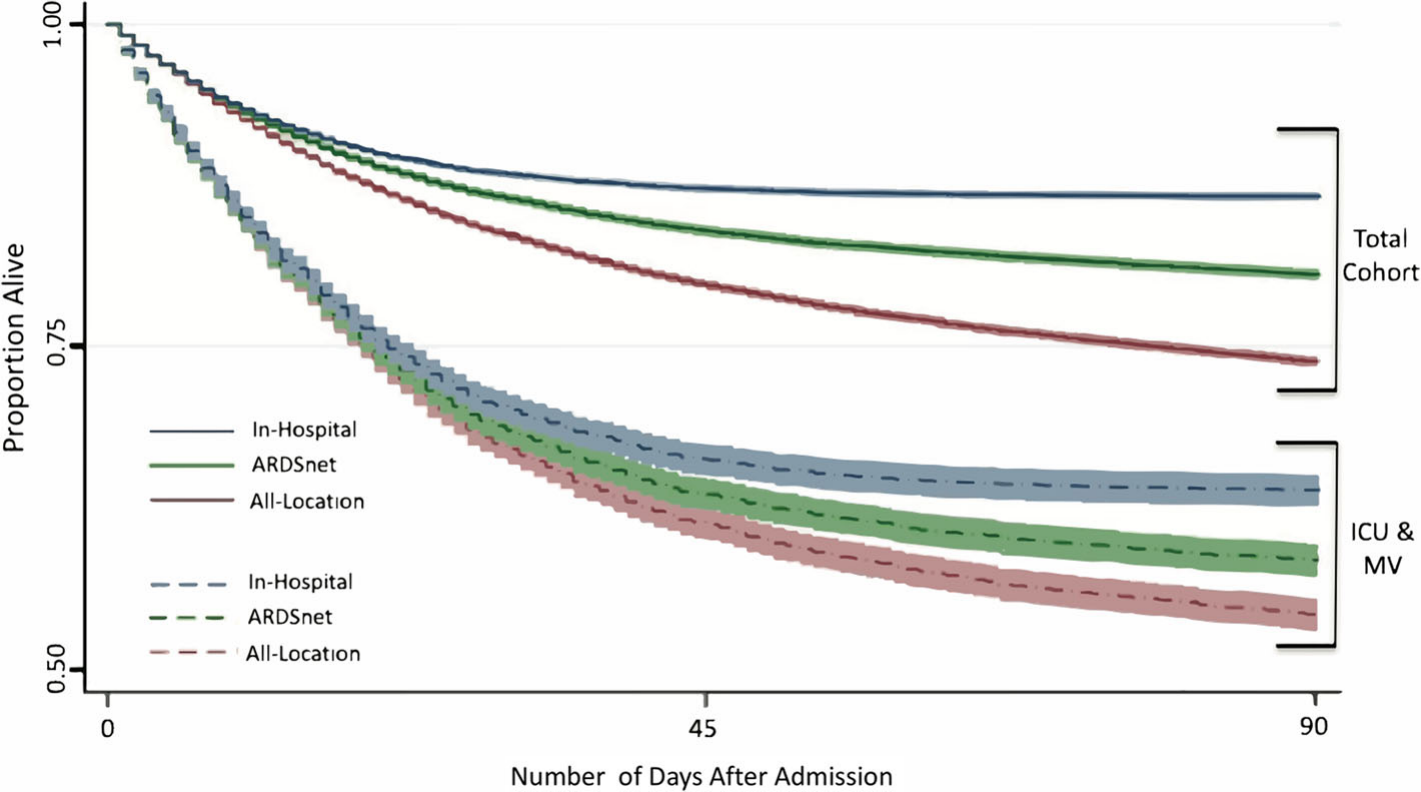
Survival curves for mortality endpoints, with 95% confidence intervals. *ICU* intensive care unit, *MV* mechanical ventilation. 90-day all-location mortality: death within 90 days of randomization at any location. 90-day ARDSnet mortality: death in a healthcare facility of greater intensity than the patient was in prior to the hospitalization during which they were randomized. 90-day in-hospital mortality: death prior to discharge from the primary hospitalization and within 90 days of randomization

For the total cohort, 8111 experienced all-location 90-day mortality (26.4% [95% CI 25.9–26.9%]), 5904 experienced ARDSnet 90-day mortality (19.2% [95% CI 18.8–19.7%]), and 4107 experienced in-hospital 90-day mortality (13.4% [95% CI 13.0–13.8%]) (Fig. 1). All pairwise comparisons were significantly different (*p* < 0.001). In order to detect a 20% relative reduction in mortality with 80% power and a 5% false positive rate, an RCT using an all-location 90-day endpoint would require 2080 patients. ARDSnet 90-day mortality would require 3080 patients, and in-hospital 90-day mortality would require 4796 patients. The results of our secondary analysis are reported in Additional file 1.

Differences in event rates persisted after stratifying patients by inpatient location and mechanical ventilation status (Table 2). For example, 5280 patients received both ICU care and mechanical ventilation. In that subgroup, 2438 experienced all-location mortality (46.2% [95% CI 44.8–47.5%]), 2181 experienced ARDSnet mortality (41.3% [95% CI 40.0–42.6%]), and 1894 experienced in-hospital mortality (36.0% [95% CI 34.7–37.3%]) (Fig. 1).

**Table 2:** Number of deaths, mortality rates, and sample sizes for each mortality outcome: stratified by location and ventilation status. Sample size power calculations were those required to a 20% relative reduction in mortality, with 80% power and a 5% false positive rate

		In-hospital	ARDSnet	All-location
		mortality	mortality	mortality
Overall cohort (*N* = 30,691)	# of deaths	4107	5904	8111
	Mortality rate (%)	13.4	19.2	26.4
	Sample size	4796	3080	2080
No ICU stay (*N* = 17,964)	# of deaths	1025	2043	3396
	Mortality rate (%)	5.7	11.4	18.9
	Sample size	11,756	5560	3100
ICU stay, no MV (*N* = 7447)	# of deaths	1188	1680	2277
	Mortality rate (%)	16.0	22.6	30.1
	Sample size	3778	2488	1710
ICU stay & MV (*N* = 5280)	# of deaths	1894	2181	2438
	Mortality rate (%)	36.0	41.3	46.2
	Sample size	1326	1074	892

90-day all-location mortality: death within 90 days of randomization at any location. 90-day ARDSnet mortality: death in a healthcare facility of greater intensity than the patient was in prior to the hospitalization during which they were randomized. 90-day in-hospital mortality: death prior to discharge from the primary hospitalization and within 90 days of randomization. All pairwise comparisons of event rates statistically significant at *p* < 0.05

*MV* mechanical ventilation, *ICU* intensive care unit

When calculating sample sizes for studies of mechanically ventilated patients, all-location 90-day mortality required the smallest sample size: 892 patients. ARDSnet 90-day mortality would require 1074 patients, while in-hospital 90-day mortality would require the largest sample size of 1326 patients.

## Discussion

In this study of Veterans hospitalized for sepsis, we observed important differences in the sample size required for various 90-day mortality outcomes. All-location mortality required 57% fewer patients to achieve the same power compared to in-hospital mortality. In other words, an RCT would have to suffer a 28% loss to follow-up rate in each arm to negate the difference between overall and in-hospital mortality. These findings persisted even in sicker patients. In mechanically ventilated ICU patients, all-location 90-day mortality required 32% fewer patients compared to in-hospital mortality. These results have implications for sepsis trialists who seek to gain the most statistical power while facing limited budgets.

The conceptual limitations of in-hospital mortality have been well described for decades [17]. However, our study uniquely compares in-hospital mortality with other definitions from a quantitative standpoint and notes a significant impact on event rates. This confers substantial variability in the power achieved for a given sample size. We demonstrate that an RCT could almost double its measured event rate by using all-location 90-day mortality versus in-hospital mortality. All-location mortality may be particularly important for sepsis trials given the high rate of ongoing mortality in patients surviving hospitalization—late deaths not explained by patients’ preexisting health status, but rather the lasting effects of sepsis [9, 11].

There are potential budget implications of variable event rates on RCT planning. Recent data have demonstrated rapidly increasing RCT costs, with patient recruitment being a primary factor [1]. Efficient allocation of resources requires balancing multiple variables, including both patient recruitment and retention, required sample size, appropriate primary outcome, and reliable event rate measurement. The complexity of these issues could be somewhat mitigated by precise, quantitative information [18–20]. Pilot data on outcome event rates would provide this information.

The results of this study have implications for the trial design process at large, though more from a methodological perspective. Previous critical care RCTs demonstrate different degrees of event rate variation across outcome definitions. This makes better characterization of this variation *a priori* important for power calculations. For example, the CHEST trial noted an ICU mortality of 10.9%, an in-hospital mortality of 14.2%, and an overall mortality of 17.4% at 90 days [21]. However, the CATS trial found almost identical rates of mortality at 90 days for in-hospital vs overall: 50.3% vs 51.2% [22]. These differences are likely not just a product of case-mix variation, but also related to context-specific factors, such as alternative care pathways and variable healthcare system structures. Therefore, translating event rate measurements between patient populations and contexts will lead to imprecise estimates. This makes context-specific pilot work in the anticipated sample of an upcoming trial of value to ensure optimal accuracy of sample size calculations.

There are limitations to this study. First, there are factors that impact power calculations which were not accounted for in our study, such as loss to follow-up. This would necessitate an upsizing of the required sample for all-location compared to in-hospital mortality. Second, it is unclear whether a therapy for sepsis patients will have efficacy out to 90 days post intervention. However, the increased post-discharge mortality for sepsis patients makes it logical that an effective sepsis therapy could impact both short-term and long-term outcomes [9]. Finally, there are limitations to administrative claims as a source for identifying hospitalized patients with sepsis, including misclassification of patients. In this study, we used the method of Angus et al., which has been shown to identify a sample of predominantly patients with severe sepsis, with some limitations [23, 24].

## Conclusions

Sample size calculations are an integral component of RCT design. It has been theoretically understood that these calculations are impacted by the varying event rates of different outcomes. However, the precise magnitude of this impact has not been described in clinically relevant populations. Our study empirically demonstrates substantial differences in required sample sizes by simply varying the location where death was defined. This suggests that the potential implications may warrant careful pilot work during RCT design. These differences between outcomes have practical implications for trialists with respect to budgeting, resource allocation, and logistical planning of RCTs.

## Additional file

Additional file 1: Results for power calculations when using absolute risk reduction. (DOCX 19 kb)
