# Supplementary material for: Sample size implications of mortality definitions in sepsis: a retrospective cohort study
Source: Trials. 2018 Mar 27;19:198. doi: 10.1186/s13063-018-2570-2 (PMC5870299; doi:10.1186/s13063-018-2570-2)
Supplement: Supplementary file 1 — Results for power calculations when using absolute risk reduction. (DOCX 19 kb) [file 13063_2018_2570_MOESM1_ESM.docx]

**Additional File: Results for Power Calculations When Using Absolute Risk Reduction**

**Table S1**: Number of Deaths, Mortality Rates, and Sample Sizes for Each Mortality Outcome: Stratified by Location and Ventilation Status. Sample size power calculations were those required to detect a 5% absolute reduction in mortality, with 80% power and a 5% false positive rate ^*^

|  | In-Hospital  Mortality | ARDSnet  Mortality | All-Location  Mortality |
| --- | --- | --- | --- |
| No ICU Stay (N=17,964) | # of Deaths = 1,025  Rate = 5.7%  Sample Size = 388 | # of Deaths = 2,043  Rate = 11.4%  Sample Size = 1,016 | # of Deaths = 3,396  Rate = 18.9%  Sample Size = 1,720 |
| ICU Stay, no MV (N=7,447) | # of Deaths = 1,188  Rate = 16.0%  Sample Size = 1,466 | # of Deaths = 1,680  Rate = 22.6%  Sample Size = 2,016 | # of Deaths = 2,277  Rate = 30.1%  Sample Size = 2,508 |
| ICU Stay & MV (N=5,280) | # of Deaths = 1,894  Rate = 36.0%  Sample Size = 1,398 | # of Deaths = 2,181  Rate = 41.3%  Sample Size = 2,980 | # of Deaths = 2,438  Rate = 46.2%  Sample Size = 3,088 |

*MV: mechanical ventilation; ICU: intensive care unit. 90-day all-location mortality: death within 90 days of randomization at any location. 90-day ARDSnet mortality: death in a healthcare facility of greater intensity than the patient was in prior to the hospitalization during which they were randomized. 90-day in-hospital mortality: death prior to discharge from the primary hospitalization and within 90 days of randomization. All pairwise comparisons of event rates statistically significant at P < 0.05

**Additional File Results and Discussion***:* In this alternative analysis, differences in sample sizes persisted. However, the relationship was different. In order to detect a 5% absolute reduction in mortality with 80% power and a 5% false positive rate, an RCT using all-location 90-day endpoint would require 2,256 patients. ARDSnet 90-day mortality would require 1,728 patients, and in-hospital 90-day mortality would require 1,178 patients. Differences in event rates persisted after stratifying patients by inpatient location and mechanical ventilation status (**Table S1**).

Variation in sample sizes in this supplemental analysis revealed a different relationship compared to the primary analysis. The principal rationale rests with the fact that absolute risk reduction (ARR) as a measure of treatment effect is more highly correlated with baseline event rates than relative risk reduction (RRR) (1,2). In other words, ARR accounts for both treatment efficacy and baseline event rates. This increased correlation adds complexity to power calculations because variation in event rates will mathematically impact ARR as a measure. For example, this can be seen in those patients who did not require an ICU stay. In-hospital mortality had a baseline event rate of 5.7%. A treatment applied here with an ARR of 5% lowers the event rate to 0.7%, which is an RRR of 87.7%. This is compared to all-location mortality, where the RRR is 26.4%. In most circumstances, a treatment with an RRR of 87.7% is more effective than a treatment with an RR of 26.4%, despite the fact that the ARR is the same. This results in a decreased sample size necessary to adequately power a trial. Thus, the differences noted in the supplemental analysis are multifactorial: variation in event rates, and variation in treatment efficacy.

1. Schmid CH, Lau J, McIntosh MW, Cappelleri JC. An empirical study of the effect of the control rate as a predictor of treatment efficacy in meta-analysis of clinical trials. *Stat Med* 1998; 17: 1923-1942.

2. Furukawa TA, Guyatt GH, Griffith LE. Can we individualize the 'number needed to treat'? An empirical study of summary effect measures in meta-analyses. Int J Epidemiol 2002; 31: 72-76. PMID 11914297
